# Beyond fluency: Exploring language acquisition and personal growth of students studying abroad

**DOI:** 10.12688/openreseurope.20060.1

**Published:** 2025-11-20

**Authors:** Rezarta Ramadani, Sejdi Sejdiu, Veronika Sufaj

**Affiliations:** 1 English Language and Literature, University of Prizren, Prizren, Kosovo, 70000, Kosovo (Serbia and Montenegro)

**Keywords:** Study abroad, English language, academic development, intercultural competence

## Abstract

This study explores the intellectual and personal development of English Language students in study abroad programs, with an emphasis on the benefits of immersion in the target country. Thirty students from the University "Ukshin Hoti" Prizren, enrolled in graduate and undergraduate studies abroad, were interviewed and surveyed using questionnaires to show their attitudes and experiences. The findings show that immersion helped significantly in acquiring the language. This is consistent with the fact that most of these students, having begun learning English at primary level and self-assessed themselves as already being well-equipped before studying abroad, further built a solid foundation through immersion to excel in English. Studying abroad was regarded as a gateway to career success, and as making them more marketable in the global employment market by providing them with bilingual competence and intercultural awareness that is needed in workplaces that are ever more globalized. The case study informs an understanding of how study abroad provision can have an influence on students' future engagement with the language. Participants reported substantial improvement in their capacity to think in English more directly rather than translating from Albanian - a measure of the effect of study abroad on language learning and individual transformation.

## Plan language summary

This study looks at how studying in another country helps students improve their English and grow personally. Researchers focused on students from the University "Ukshin Hoti" Prizren who traveled abroad for their studies. Through interviews and surveys, students shared their experiences of learning English in an immersive environment.

The results showed that being surrounded by English speakers made a big difference in their ability to learn the language. Many of these students had already started learning English in primary school and felt confident before going abroad. However, living in an English-speaking country gave them even more opportunities to practice and improve.

Beyond language skills, students saw studying abroad as a stepping stone for career success. They felt that their ability to speak multiple languages and understand different cultures made them more attractive to employers, especially in today's global job market.

One key takeaway from the study was that students reported thinking in English more naturally, without needing to translate from Albanian. This shows how deeply studying abroad can shape language learning and personal growth.

In short, studying abroad not only helps students master English but also prepares them for better job opportunities and a more connected world.

## Introduction

The practice of studying in foreign countries has an immense and rich historical context, dating back to Aristotle, and has always been seen as a prized way of advancing cultural, educational, and intellectual development ( İlhan & Şahin, 2020). In different geographic locations and communities, educators have encouraged people to experience different settings to enable personal and intercultural development. While studying abroad can be traced in earlier times in history, the phenomenon became more evident in the nineteenth century with a record increase in the number of students going abroad for studies. Currently, international study experiences provide students with unique learning opportunities that are both comprehensive and deep. Through these experiences, students engage in a myriad of cultural experiences, develop key life skills such as flexibility and autonomy, and improve their professional skills.

Professional training in the current world of the global workforce is no longer sufficient to gain employment. Employers increasingly require more attention to communication skills, problem-solving, and team building ( Sisavath, 2021). Global studies play the role of providing the required skills to the student while fostering multilingualism and respect of diversity among the cultures. Multilingualism is required to bridge the people from the cultures and promote international educational activities. Global studies equip the student with the capacity to learn the target language and culture and maximum exposure to native speakers that promote fluency and vocabulary building.

The global number of international students has continued to increase significantly in recent decades. More than six million students were reported to be studying abroad by the year 2019, with a consistent rise from previous decades ( UNESCO Institute for Statistics, 2020). Although previous estimations had projected 8 million students by the year 2025 ( Kritz, 2012 as cited in Nghia, 2019), the COVID-19 pandemic and other global events would have potentially impacted such projections. In spite of this, the United States, United Kingdom, Australia, Germany, and France remain the main host nations of international students ( British Council, 2019; OECD, 2023).

This expansion mirrors the increasing recognition of study abroad programs as vital for obtaining global competencies, intercultural communication abilities, and advanced education. Recent research shows diversification in destinations as more and more countries are investing in international student attraction via scholarships and collaborations. Such trends emphasize the sustaining applicability of study abroad opportunities in shaping worldwide education and career pathways.

## Enhancing higher education in Kosovo through EU-funded programs

European Union (EU) programs have also benefited the education system in Kosovo through international collaboration under Erasmus+, Comenius, Tempus, and One for Youth programs. Erasmus+, launched in 1987 as a flagship program for mobility and cooperation in European higher education, has been especially successful.

It is the EU program that supports areas of education, training, youth and sport in Europe and neighboring regions. It has an estimated budget of €26.2 billion for the period 2021–2027, which is nearly double the funding compared to its predecessor program (2014–2020). Kosovo, as part of the Western Balkans region, is considered a priority region for cooperation with the EU, within the Erasmus+ program with a total budget of €374 million for the period 2021–2027.

Kosovo Erasmus+ Office (KEO) is the focal point for all actors involved in the Erasmus+ Programme in the area of Higher Education, Vocational Education and Training (VET), Youth and Sport ( Kosovo Erasmus+ Office, 2024).

Between 2015 and 2020, a total of 4,177 Kosovo students and staff were financed by Erasmus+ with International Credit Mobility scholarships - a significant increase from just 331 participants back in 2012 ( Kosovo Erasmus+ Office, 2024).

In the last five years, the Erasmus+ program has been instrumental in defining the international higher education experience for Kosovar students. Despite the difficulties, student mobility has been robust and has registered notable growth. Student mobility fell sharply in 2020 with the COVID-19 pandemic since travel bans and uncertainty meant fewer students traveled outside the country to study. In 2021, with an improvement in the global situation, there was a modest recovery with more students returning to plans of international study. In 2022, the program saw a strong recovery in participation, echoing the willingness of students to avail themselves of Erasmus+ opportunities following a period of uncertainty. The trend continued in 2023, with mobility figures returning to and even exceeding pre-pandemic levels. In 2024, Kosovo students have shown even more enthusiasm for studying abroad, echoing the increasing popularity of international education. On the destination front, some European nations have forever been the most favored destinations.

Germany and Austria are still the most desirable destinations among Kosovar students, given their high standards of education and scholarships. Italy is next, especially for students of humanities and social sciences. Turkey is also a much-desired destination due to its cultural and historical connections with Kosovo. Poland has grown more popular in the last several years due to its diverse study options, while France and Spain have also become favorite destinations that have pulled students who wish to study arts, technology, and languages. In general, the last five years have seen a growing interest in foreign education among Kosovar students. The consistent growth in the number of outgoing students indicates not just the success of the Erasmus+ program but also the growing significance of international academic mobility. With opportunities continuing to expand, the trend is most likely going to persist, further solidifying Kosovo's integration into the wider European educational space ( Kosovo Erasmus+ Office, 2024).

## The local context

This paper reflects on the importance of living or studying in a target language country and the changes that a student undergoes as an individual, and how it may affect their future interaction with the language.

The project is located at the University “Ukshin Hoti” Prizren (UPZ) in young the Republic of Kosovo. Founded in 2010, UPZ is the second-largest university in Kosovo with a long tradition of multilingualism and multiculturalism, typical of the West Balkan region.

The University of Prizren (UPZ) is one of the few officially trilingual European universities, with others being Bolzano, Fribourg, Lleida or Luxembourg. As in all of them, the multilingual character of the university is first and foremost designated to serve the needs of a multilingual local population, while trying to cater to the pressures for internationalization in higher education. In addition to representing the local communities, UPZ follows the global trend of increased internationalization offering study programs in three local languages (Albanian, Bosnian and Turkish) and in two foreign languages (English and German).

Thus, UPZ has increased the internationalization of academic staff and students, curricula, study programs and research, through, e.g. membership in international associations and quality assurance standardization, international academic and research networking, mobility as well as establishing a university international profile with attractive study programs.

Study abroad is rightly established as one of the gateways to language acquisition. Immersion provides wide scope for native speaker contact and target culture exposure. Students are granted greater fluency and lexis while becoming interculturally competent.

The main aim of this study was to investigate the value of studying abroad for English programs at various universities by asking the following questions:

1. How do the situations and experiences that students are exposed to while away from home affect their ability to learn a second language?
2. How does a student's personal development while studying abroad affect second language acquisition and their future career?

## Literature review

Language learning and retaining a foreign language is an essential part of study abroad programs, especially if the language of the host country differs from that of the students. Becoming exposed to a new culture renders the students global learners, with a great deal of research confirming that studying abroad accelerates language acquisition and increases language proficiency ( Isabelli-Garcia, 2006). Although the learning capacity of a language is different according to internal and external variables, it is always true through research that greater exposure to native speakers hastens the process of learning. Even the less capable learners can convey meaning through compensatory strategies ( Rababah, 2002). Interaction is also part of enhancing communication skill **s** and language learning, based on Segalowitz *et al.* (2004). Students are more confident in specific interactions, and the intensity of exposure to the target language may lead to a deeper conversation that acquires a higher level of proficiency, such as engaging in conversations, exchanging impressions, and feelings in the target language ( Dwyer, 2004). Learners have maintained proficiency and overall language development due to continuous engagement with host settings.

In contrast to the myth that natural exposure is sufficient for second-language acquisition ( Isabelli, 2001), there is clear evidence that intentional contact with host nationals and cultures significantly improves confidence and competence. Segalowitz *et al.* (2004) indicated that study-abroad students developed advanced narrative competence, improved conversational proficiency, and relied less on compensatory mechanisms. These interactions enable students to participate in higher-level discussions and develop more sophisticated language ability, with ongoing interaction promoting intercultural competency in conjunction with linguistic ability. Most studies on studying abroad emphasize the students’ linguistic and language improvements. Various data and interviews advocate that studying abroad has a beneficial effect on overall academic achievement ( Cardwell, 2020). Academic achievement is understood as a broader educational experience that increases confidence and language acquisition.

Though language learning is generally the focus of study abroad research, it has an influence on academic performance, personal development, and professional opportunities. Study abroad facilitates language retention, intercultural proficiency, and employability ( Kinginger, 2011). Pinar (2016) illustrated that the students developed fluency, speech rate, grammatical accuracy, pronunciation, and vocabulary while abroad. Study abroad programs also consolidate morpho-syntactic forms and promote independent learning ( La Sala, 2018). While at home, students might perform better in terms of grammar and vocabulary learning, their counterparts abroad develop greater communicative competence through real interaction ( Collentine, 2004). Albanian students tend to find English pronunciation difficult because of unknown sounds ( Alimemaj, 2021).

DeKeyser (1991) did not find differences in grammar or fluency between study-abroad and home learners but reported varied interaction patterns resulting in higher proficiency among study-abroad learners. U.S. and European students who exchanged also showed higher intercultural communication competence through frequent contact with local communities ( Williams, 2005). Furthermore, regular interaction with native speakers helps learners be aware of their output and that of native speakers allowing them to adjust their language in response to native speakers’ statements ( Hernàndez, 2018). These contacts enable students to tune their language use while mitigating loneliness and acculturative stress through cross-cultural interaction ( Smith & Khawaja, 2011).

Yet, less contact with locals or mentors can be obstructive to academic success and adjustment. Kılınç *et al.* (2020) highlighted that less contact typically leads to discomfort and poor academic performance. The length of programs is also a decider of linguistic and cultural change; the longer the period, the more advantageous it becomes. Dwyer (2004) established that one-year programs allow for greater independence and cultural comprehension relative to shorter durations.

The personal development that occurs with study abroad has become ever more acknowledged by scholars and business leaders. Contemporary research demonstrates that participants repeatedly register greater self-confidence, flexibility, and global-mindedness following study abroad. Sisavath (2021) elucidated how study-abroad programs enhance professional competency, academic performance, international capacity, and professional opportunities. Yang (2017) cited how bilingual education expands the global mobility and professional opportunities of university students. Franklin's (2010) findings suggested that foreign study graduates are more likely to work in the international community due to their intercultural skills.

Despite such benefits, some studies exhibit conflicting findings regarding employability. Di Pietro (2019) demonstrated that students who had not studied abroad were more employed than their counterparts who had studied abroad. There might be several reasons for the drawback that study abroad students might have, for example, not all graduates might transfer their gained knowledge overseas into something that companies value ( Di Pietro, 2019). Some studies revealed how interested companies are in applicants who have studied abroad. Data gathered from UK companies revealed that graduates with international study experience are more employable ( Fielden, 2007). Most of the participants of Kılınç *et al.* (2020) saw studying abroad as a way to more prominent and well-paying employment which led to a greater future. Another case study proves that differences in wages were clear based on the location of the study abroad program, Florence participants had higher wages than non-study abroad participants ( Posey Jr, 2003). However, Nghia (2019) claimed that most of the students aim to find a place to work, on the other hand, there happen to be students who are willing to return home for various reasons, which is seen as an opportunity upon returning home. Studying abroad offers students to develop various and unique employability skills and competencies.

## Research design and research methods

This case study explores English language development among students in the English Department at the public university of Prizren, "Ukshin Hoti," who studied English abroad. To achieve this, a mixed-method approach using interviews and questionnaires was employed, as student perspectives were crucial to the research. The study examines whether studying abroad enhances language acquisition and its overall impact.

A mixed-method approach was used to collect and analyze both quantitative and qualitative data. The quantitative data was gathered through a questionnaire with closed-ended questions, while qualitative data was obtained from open-ended questions and interviews. Undergraduate and graduate students studying English abroad shared insights on their language development. The results were analyzed and compared to draw conclusions.

## Data collection instruments

Two instruments were used: a questionnaire and individual interviews. The questionnaire, created in Google Forms, contained 20 questions focused on exposure, duration, and language development. Interviews included nine main questions to assess participants' language progress. Conducted via Google Meet, interviews were recorded with participant consent. Data collection occurred in two phases: December 2024–January 2025 and June–July 2024. Before distributing the questionnaires and conducting the interviews, a written consent form was filled by all of the participants.

## Participants

Participants were students from the English Language and Literature Program at the University “Ukshin Hoti” in Prizren who studied English abroad. A total of 30 randomly selected students participated in the questionnaire (11 males, 19 females), with 20 in graduate and 10 in undergraduate programs as part of student exchange or mobility programs. Sixteen of these students also took part in interviews. The participants, aged 18 to 39, studied in various European countries, and both data collection methods were conducted online.

## Data analysis

A mixed-method approach was used for analysis. Quantitative data from 15 closed-ended questionnaire questions were statistically analyzed using Google Forms. Qualitative data from open-ended responses and interviews were analyzed using MaxQDA software. Interviews, consisting of nine main questions, were recorded, transcribed, and coded. Direct quotes reflecting participants' views were selected, ensuring the most compelling responses were included in the analysis.

## Results - Demographic and background information

Language development is an ongoing process, making progress difficult to measure. Among participants, 83.3% began learning English in primary school, 10% in secondary school, and 3.3% after high school. Before studying abroad, 60% believed they had strong English skills, 16% felt competent, and the rest rated themselves below that. These students studied English Language and/or Literature in Germany, Greece, England, North Macedonia, Ukraine, Turkey, Austria, the USA, and Italy. The vast majority (28 out of 30) spent 2 to 4 years abroad, while two remained for 6 to 10 years, completed postgraduate studies, and now work there. About 26.7% lived with host families, while the rest stayed in private apartments or dormitories.

The primary goal of studying abroad was to gain language competence and communication skills:

*"To become fluent in English, obtain an international degree, and secure an internship or job."*

Personal growth was also a key motivation:

*"To step out of my comfort zone, gain independence, and engage with the local community."*

Most students felt cultural differences affected their experience:

*"Socialization, setting personal boundaries, and classroom communication were different, including how professors were addressed."*

When asked about their motivation to study English after going abroad, responses were overwhelmingly positive:

*"Living with international students pushed me to study non-stop."*

Regarding pronunciation challenges, 56.7% reported no issues:

*"I struggled more with academic vocabulary than pronunciation."*

Others found native speakers difficult to understand and lacked confidence in speaking fluently:

*"I doubted my skills, even though I knew English well."*

Common language difficulties included grammar mistakes (e.g., assigning gender to nouns, as in Albanian), vocabulary gaps, unfamiliar slang, mispronunciation, and lack of cultural knowledge.

At the time of the study, 14 participants (46.7%) rated their English proficiency as “very good,” 14 (46.7%) as “excellent,” and two (6.7%) as “good.”

Studying abroad is widely seen in Kosovo as a pathway to a secure career—96.7% of participants agreed, with only one dissenting.

When asked how studying abroad improved their language skills:

- 80% reported better oral fluency
- 76.7% improved pronunciation
- 73.3% increased speech speed
- 70% expanded vocabulary
- 63.3% enhanced grammatical accuracy

On whether they would study abroad again, most said yes:

*"I’d be more open to discussions, more confident, and adapt better to the culture."*

One participant, however, stated:

*"While it helped my language skills, the experience was personally and academically difficult. I wouldn't do it again."*

## Students’ results from interviews

Sixteen interviews were conducted online via Google Meet, with participants from the questionnaire. Interviews aimed to:

1. Gain deeper insights into the language competence of University "Ukshin Hoti" students who studied English abroad.
2. Identify employers' perspectives on whether studying abroad improves job prospects.

## Impact of studying abroad on language competence

When asked about language gains, most participants reported improvements in pronunciation, vocabulary, and fluency, along with enhanced critical thinking, time management, and intercultural communication skills:

*"Confidence in speaking, using idioms, and understanding native speakers improved significantly."*

*"I developed intercultural communication skills and now speak English more fluently."*

Some, however, felt their home country already provided sufficient English learning opportunities:

*"I progressed in English even in my home country, so studying abroad didn’t make a major difference."*

## Personal growth and career gains

Students reported academic and cultural benefits from studying abroad:

*"A new perspective on teaching, a different culture, and more professionalism."*

However, one participant found little difference:

*"Our academic approach differs, but I didn’t gain much beyond new referencing styles."*

## Employability

Most participants were employed, though less than half had worked abroad. They credited their English skills for opening international job opportunities and higher salaries:

*"English is the foundation of my career—it enabled me to study abroad and secure multiple job offers."*

*"Being bilingual boosts employability, confidence, and income potential."*

## Intercultural competence

Most agreed that bilingualism enhanced global awareness and adaptability:

*"Language bridges communication gaps, helping us better understand different cultures."*

A few disagreed, emphasizing that cultural factors, not just language, drive acceptance:

*"Language paves the way, but understanding and acceptance require deeper intercultural experiences."*

## Broader influences

Many felt studying abroad shaped their mindset, career priorities, and adaptability:

*"I now view life differently, interact more openly, and make decisions with a broader perspective."*

One participant, however, reported no impact due to a demanding academic schedule:

*"I was too busy with coursework to engage in other cultural activities."*

## Advice for future students

Most encouraged studying abroad, highlighting personal and professional growth:

*"It’s a once-in-a-lifetime experience—take the opportunity without fear."*

One participant advised choosing destinations carefully:

*"Pick a country open to international students; smaller cities may pose challenges in integration and employment."*

## Discussion of findings

The findings provide valuable insight into the educational, linguistic, and personal growth experiences of the English Language students studying abroad. The findings reveal that involvement in an environment in which the target language is used considerably improves linguistic skills, with 80% reporting an improvement in oral fluency, 76.7% reporting improvement in pronunciation, and 70% reporting an improvement in vocabulary span. This outcome is supported by the findings of earlier research (i.e., Lafford & Collentine, 2006), which highlights the significance of immersion in the development of fluency and vocabulary. However, the challenge with grammar and understanding native speakers indicates that language acquisition does not follow uniformly across all aspects. This indicates that while immersion can speed up specific aspects of learning the language, the need for formal support with grammar and academic language persists. Particularly, 56.7% reported having no problems with pronunciation, yet others were troubled with native accents and idiomatic expressions. Such differences make it necessary to have specific approaches to teaching the language both before and during the period abroad to cater to the specific needs of every learner.

The findings also highlight the profound impact of international study on personal growth. Most participants stressed the value of going beyond comfort zones, becoming self-sufficient, and engaging with host communities as basic tenets of personal development. The findings support Dwyer's (2004) findings, which established that the duration of international study abroad increases autonomy and adaptability indicators. Cultural differences, however, were challenging for many of the participants, particularly regarding social relations and communication in the school setting. This is supported by Smith & Khawaja's (2011) findings that cultural adjustment usually happens over time and is influenced by interactions with host societies. Prioritizing the teaching of intercultural communication skills as an important goal means study abroad programs should include cultural orientation courses to increase understanding and coping with these differences.

Motivation emerged as a significant factor in language acquisition and overall success during the study abroad period. Statements such as "Living with international students pushed me to study non-stop" highlight the positive influence of peer pressure and international environments on academic performance. However, some participants expressed doubts about their abilities despite measurable improvements, reflecting the role of self-confidence in language learning.

Additionally, one participant noted that the experience was academically demanding to the point of limiting cultural engagement. This finding suggests that balancing academic workloads with opportunities for cultural immersion is crucial for maximizing the benefits of studying abroad.

Motivation emerged as an important variable in both language learning and general achievement throughout the study abroad experience. Statements like "Studying with international students made me work to the best of my capacity" highlight the positive role played by international settings and peer modeling towards acquiring education. However, many participants expressed doubt about the capabilities, even when seen to be making improvement, thereby highlighting the imperative for self-confidence to form part of the learning process.

In addition, one participant reported that the intense academic rigor of the experience limited the availability for cultural participation. This observation suggests the need to balance academic commitments with potential for cultural contact to realize the benefits of studying abroad.

The participants uniformly stressed the importance of having proficiency in the English language to enhance career prospects, correlating global employment prospects and work self-confidence with experiences in global learning. Such findings support Sisavath's (2021) claims, which highlighted the career advantage that comes with gaining bilingualism and intercultural capabilities.

It is notable that less than half of the respondents had worked abroad after completing their studies. This raises a question regarding the possible systemic barriers to international employment opportunities and whether additional career guidance services could successfully resolve this imbalance.

The findings show the need for increased support around the study abroad programs offered by universities. While students overwhelmingly viewed international study as transformative, they simultaneously identified certain areas for maximized institutional support, such as prior to departure language preparation, cultural acclimatization, and academic advising.

Given the overwhelming agreement (96.7%) among the participants regarding the significance of international learning experiences for professional development, it is necessary for educational institutions to consider increased structured inclusion of such programs in the curriculum. Additionally, the removal of cost barriers could make such experiences more accessible to more students.

## Conclusion

The study highlights the profound impact of studying abroad on the students` academic, linguistic, and personal development toward achieving English fluency. Being exposed to an environment with plenty of interaction in the language greatly enriched the participants' oral fluency, pronunciation, vocabulary, and grammaticality, while also fostering intercultural communication skills and critical thinking skills. Additionally, studying abroad provided students with many channels for personal growth, including increased autonomy, flexibility, and broader outlooks. The findings shed light on the double potential of studying abroad: the development of language skills and the acquisition of global skills so vital in the globalized community.

However, cultural adjustment problems, grammatical competency, and doubts about effective communication underscore the need for increased and structured assistance for students both during and prior to international education experiences. Despite such concerns, the overall positive feedback received from the participants stresses the importance of incorporating the programs for studying abroad in the curricula at universities to effectively prepare the students for global career activities and cross-cultural interaction.

## Recommendations

Future research should focus on investigating the long-term impact of studying abroad on language learning and intercultural understanding. Longitudinal research designs can track students years after their studying abroad to assess the development in their linguistic skills, intercultural adaptability, and career trajectory throughout the years. More importantly, comparative studies across different host countries and different curricula can provide more insight into how different contexts influence learning languages and personal growth. Research can also be conducted to see whether the outcomes of study abroad programs are affected by individual differences in motivation, personality, and initial language level.

Another area of investigation involves the impact of program duration on cultural and academic integration among students. While this study highlights the benefits related to 2–4 years abroad, research can be conducted to see if shorter or more extended periods result in similar or different outcomes. Moreover, the examination of the challenges facing students in small city settings or areas with weak international integration can make it easier to develop measures for promoting integration and support arrangements in such settings.

Future studies can explore the career paths related to the benefits acquired through international learning in different fields. Evaluating the degree to which similar benefits exist in other than the field of English Language and Literature can make it possible for the benefits of international learning to be thoroughly evaluated. Additionally, comparing the economic barriers and overall economic feasibility for international learning can have the potential to inform policy aimed at increasing accessibility for students with different backgrounds. Finally, exploring the role that technology can play in augmenting traditional study abroad programs, such as through virtual exchange programs and online language learning software, is particularly relevant for students who are facing barriers to studying abroad. Such studies can build on the findings presented here to make study abroad programs more efficient and feasible to implement, providing actionable suggestions for universities worldwide.

## Limitations of the study

Some limitations must be considered in the study’s conclusion. Future studies are encouraged to investigate further the importance of studying abroad as an important part of language acquisition by assessing longitudinal research to prove findings by assessing students from the beginning of the journey up to the end of their studies. The questionnaire and interview were conducted only to the English language students from the University ‘Ukshin Hoti’ in Prizren who continued their studies abroad. Out of 73 students, only 30 of them responded to the questionnaire. The interview was conducted with 16 students only. Consequently, the results may not be generalized for all English language students who continued their studies abroad from Kosovo. Moreover, these findings may show bias since they are based on self-reported information.

Based on the findings of this study, more research is needed to incorporate students in their home country to compare and contrast the development of students who are or were abroad to improve the identification of the results of study abroad participation. This study does not probe into students’ motivations related to language learning or their perceptions of the environment.

## Ethics and consent

This study was conducted in accordance with the principles outlined in the Declaration of Helsinki. Ethical approval for this research study was obtained from the Scientific Committee of the University of Prizren on 12.05.2024. All participants provided written informed consent prior to participation.

## Data Availability

Due to the formulation text of the consent form before this paper was written, all the data are strictly for internal usage and cannot be shared with the public, due to a specific sentence within the consent form, signed by all the participants, that states: “Data will be stored securely and will be accessible only to the research team”. According to this statement, data publication violates the code of conduct of the Scientific Council of the University of Prizren, the approver of the consent form, and the Declaration of Helsinki. Zenodo. Extended data for research paper. Extended data for research paper ( 10.5281/zenodo.16268945) The project contains the following extended data: Consent form. (information about the consent form) Questionnaire. (the questionnaire by which the data was collected) Transcription convention. (the rules based on which the interviews were transcribed).
