# Supplementary material for: Beyond fluency: Exploring language acquisition and personal growth of students studying abroad
Source: Open Res Eur. 2025 Nov 20;5:353. [Version 1] doi: 10.12688/openreseurope.20060.1 (PMC12723210; doi:10.12688/openreseurope.20060.1)
Supplement: Peer review history

# Reviewer report: Reviewer response for version 1

**DOI:** 10.21956/openreseurope.21698.r64701

**Published:** 2025-12-24

**Authors:** Le Thanh Thao

**Affiliations:** 1 School of Foreign Languages, Can Tho University, Can Tho, Vietnam

Dear Authors,

 Thank you for your manuscript. I need to be transparent that, in its current form, I am not able to recommend approval of the article.

 As a starting point, I would invite you to pause and ask: what is the one core contribution you want this article to make? Is it that study abroad improves language proficiency, that it strengthens employability, that it shapes personal development, or that Kosovo offers a special case that challenges or extends existing literature? At present, the manuscript describes all of these aspects, but it is difficult to see what is genuinely new compared with the many existing studies on Erasmus+, study abroad, and language learning. Much of the introduction and discussion restates widely known claims: immersion supports oral fluency, students grow in self-confidence and intercultural awareness, and employers value international experience. These points are true and well supported, but they are already well-established in the literature. What is it, precisely, that this particular group of 30 students from the University of Prizren allows you to say that other studies could not say? Is it something about the post-conflict context of Kosovo, about the trilingual environment, or about specific barriers and opportunities that your students face? Clarifying this would be the first crucial step in sharpening the contribution.

 Related to this, the research questions are quite broad and somewhat vague. You ask how “situations and experiences” affect L2 learning, and how “personal development” affects language acquisition and future careers. Could you define more clearly what you mean by situations, by experiences, by personal development? Are you focusing on specific aspects such as interaction with host communities, academic pressure, or institutional support? Without a clearer conceptual framework, the questions risk remaining at a very general level, and the findings then mirror that generality. You might ask yourselves: if I had to sketch a simple model on one page, showing how study abroad, personal development, language proficiency, and employability are linked in this specific context, what would that model look like? What elements would be central, and what tensions or contradictions would appear?

 There are also important methodological issues that undermine the strength of your claims. You state that 30 students were “randomly selected,” but the description of the data collection suggests a voluntary response to a Google Forms questionnaire. If students chose to participate after receiving a link, this is not random sampling in the statistical sense, but it is convenience or self-selection sampling. How exactly were participants recruited? How many students received the link, and how many responded? Clarifying this is not just a technical detail, but it shapes how far you can generalise your findings. If you present the sample as random when it is not, readers may be misled about the representativeness of your results. I would strongly recommend that you revise this description and consider carefully what kind of sampling you actually used.

 The timeline of data collection also needs careful checking. At one point, you report that data were collected between December 2024 and January 2025, and also between June and July 2024. As written, this sequence is confusing and chronologically impossible. Are some of these dates typographical errors? Which phase took place first? In a research article, even such “small” inconsistencies can raise doubts about the reliability of reporting. It might help to ask yourselves: if a reader had to reconstruct exactly when each part of the study happened, would they be able to do so from the current text?

 You describe the study as mixed-methods. However, the quantitative component remains limited to basic descriptive statistics from a small sample, and the qualitative component is only briefly described. This raises a further question: what does “mixed-methods” mean for your study in practice? For the questionnaire, you mention 20 items, of which 15 closed-ended questions are used for quantitative analysis. How were these items developed? Were they adapted from existing instruments, or created specifically for this study? How did you check their clarity and reliability? Did you consider internal consistency or any form of validation? Including sample items or an overview of the scales would help readers understand what exactly you measured when you talk about motivation, self-perceived proficiency, or perceived gains.

 Similarly, in the qualitative part, you refer to interviews, transcription, coding, and the use of MaxQDA. Yet very little is said about how the coding was actually done. What coding approach did you adopt: inductive, deductive, or a combination? How did you move from raw transcripts to the themes you present? Were there multiple coders, and if so, how did you resolve differences? Without a clearer explanation of these steps, the qualitative findings risk appearing as a collection of illustrative quotes rather than the result of a systematic analysis. You might ask: if someone wanted to replicate the qualitative analysis or build on it, would they have enough information in the manuscript to see what we did?

 A central issue concerns the way you talk about “language acquisition” and “proficiency.” Throughout the article, you use strong language to suggest that studying abroad improved students’ English, especially their oral skills and vocabulary. However, these claims rest entirely on self-reported perceptions and self-ratings. There are no pre-post tests, no objective proficiency measures, and no comparison group of students who did not go abroad. In this situation, can we really say that study abroad caused an improvement in language proficiency, or can we only say that students felt they had improved? This is not a minor distinction. Many readers will take “language acquisition” to imply measurable changes in performance, and your current design does not allow such claims. You may want to reconsider your wording: could it be more accurate to frame your findings as students’ perceptions of their language development, rather than as demonstrated gains?

 The same caution applies to employability. You argue that the experience abroad increased students’ chances in the labor market. Yet again, all the data are self-reported expectations and beliefs, not actual employment outcomes. Have you considered how social desirability, optimism, or institutional narratives around Erasmus+ might shape these responses? It may be more honest and methodologically sound to describe the findings as students’ beliefs about employability, rather than as evidence that employability has in fact improved.

 When it comes to analysis, much of the quantitative reporting consists of percentages summarising responses. This is understandable with a small sample, but it does mean that the results remain at a descriptive level. Are there patterns you could explore, even with caution, that might deepen the analysis? For example, do students who stayed longer abroad report different perceived gains from those who stayed for a shorter period? Do host country or level of study appear to shape experiences? These kinds of questions, if handled carefully, might help you move beyond “x% said this” and “y% said that” towards a more nuanced picture.

 On the qualitative side, the quotations you present are often interesting, yet they are not fully integrated into a deeper interpretive framework. Many quotes reiterate that students feel more confident, more motivated, or more open-minded. Can you identify more subtle themes, contradictions, or tensions? Were there students who did not feel significant gains, who struggled with returning home, or who felt that the international experience did not translate into concrete career opportunities? Bringing such complexities into the analysis could make the case study more compelling and distinctive.

 I would also encourage you to reflect on the strength of the link between your data and some of the broader statements in the discussion and conclusion. At times, the manuscript moves quickly from the voices of 30 students in one department to quite large claims about study abroad in general. Given the small, non-random sample and reliance on self-report, is it appropriate to speak of “profound impact” in such general terms? Could the conclusion instead emphasise what this specific case suggests, while openly acknowledging its limitations and the need for further research with more robust designs?

 For all these reasons, I am recommending that the article not be approved in its current form.

 Thank you again for the chance to engage with your work.

 With best regards,

 Reviewer

If applicable, is the statistical analysis and its interpretation appropriate?

No

Is the study design appropriate and is the work technically sound?

No

Is the work clearly and accurately presented and does it engage with the current literature?

No

Are the conclusions drawn adequately supported by the results?

No

Are sufficient details of methods and analysis provided to allow replication by others?

No

Are all the source data and materials underlying the results available?

Yes

Reviewer Expertise:

Social sciences, English education, writing research, teacher professional development

I confirm that I have read this submission and believe that I have an appropriate level of expertise to state that I do not consider it to be of an acceptable scientific standard, for reasons outlined above.

# Reviewer report: Reviewer response for version 1

**DOI:** 10.21956/openreseurope.21698.r64697

**Published:** 2025-12-22

**Authors:** Nuhi Bllaca

**Affiliations:** 1 Department of English and American Studies, University of Vienna, Vienna, Vienna, Austria

Overall, the study aims to fill an important gap in the current literature on immersion/study abroad programmes by investigating how factors such as experience of studying, working, and living abroad, and the situations students may encounter affect the acquisition and/or enhancement of English among Kosovar students. However, before the manuscript can be recommended for publication, several issues require serious attention. Below, I highlight key concerns that should be addressed in order to improve the quality and contribution of the manuscript.

**Introduction**

 The authors do not provide sufficient empirical evidence or do not elaborate it thoroughly to justify the need for the present study and, consequently, do not clearly identify a research gap in the study abroad literature that the paper addresses. The authors should explicitly state the gap, for example by highlighting the lack of research on immersion programmes in the current context (Kosovo), and/or by identifying limitations in previous studies that the present study seeks to address.

**Literature review**

 The authors cite a small number of studies on study abroad and the effects of living, studying, or working abroad on second language acquisition. However, the literature review mainly reports broad benefits of studying/living abroad without clarifying crucial details such as: (a) which language(s) participants studied, (b) which specific aspects of language development improved (e.g., fluency, vocabulary, accuracy, pragmatics), (c) the nature and amount of exposure to the target language in the host context students experienced, and (d) what challenges or constraints participants faced.

 These details matter because they help the reader understand what exactly the present study investigates and what it contributes to the current literature. They also enable meaningful comparison between prior findings and the results of the present study. Strengthening the literature review in this way would also allow the authors to articulate a more precise research gap and to explain how their study addresses it.

**Methodology**

 The authors should provide fuller information about participant characteristics, including (at minimum): participants’ L1(s), duration of stay in the host country, L2 proficiency level, field of study, and relevant background information (e.g., prior international experience). This information is necessary for interpreting the findings and assessing the study’s generalizability.

**Data-gathering instruments**

**Questionnaire:** The authors do not provide sufficient evidence of the questionnaire’s validity and reliability, which is necessary for evaluating the trustworthiness of the findings. In addition, details are missing regarding the procedures used to analyse the questionnaire data. The authors should clearly describe the analytical steps taken (and provide justification for key decisions), including how responses were coded and how themes/categories were generated (if applicable).

**Interviews:** Similar concerns apply to the interviews. The authors should specify the techniques and procedures used to analyse interview data (e.g., thematic analysis, content analysis, coding framework), describe how the analysis was conducted (including any steps taken to enhance credibility, such as double-coding or intercoder agreement, where relevant), and justify why these procedures were selected.

**Research questions**

 Although the results section suggests that the authors examined how situations, experiences, and personal development relate to English language development, the research questions do not sufficiently reflect the stated aim of exploring how such factors affect English language learning in a study abroad context. I recommend reformulating the research questions to align more clearly with the study’s aims and with the variables discussed in the results.

**Technical and referencing issues**

 In the Introduction, several claims require supporting references. In particular, statements such as the following should be supported with relevant sources:

- “Through these experiences, students engage in a myriad of cultural experiences, develop key life skills such as flexibility and autonomy, and improve their professional skills” (p. 1).
- “Global studies equip the student with the capacity to learn the target language and culture and maximum exposure to native speakers that promote fluency and vocabulary building” (p. 1).
- “In 2021, with an improvement in the global situation, there was a modest recovery with more students returning to plans of international study…”
- “In 2024, Kosovo students have shown even more enthusiasm for studying abroad, echoing the increasing popularity of international education.”

More generally, the authors should re-read the manuscript carefully and ensure that all empirical claims, trends, and generalizations are appropriately supported with up-to-date and relevant references.

If applicable, is the statistical analysis and its interpretation appropriate?

Partly

Is the study design appropriate and is the work technically sound?

Partly

Is the work clearly and accurately presented and does it engage with the current literature?

Partly

Are the conclusions drawn adequately supported by the results?

Partly

Are sufficient details of methods and analysis provided to allow replication by others?

Partly

Are all the source data and materials underlying the results available?

Partly

Reviewer Expertise:

Collaborative writing; Peer interaction; Written corrective feedback, Computer-assisted language learning, GenAI tools in language teaching and learning.

I confirm that I have read this submission and believe that I have an appropriate level of expertise to confirm that it is of an acceptable scientific standard, however I have significant reservations, as outlined above.

# Reviewer report: Reviewer response for version 1

**DOI:** 10.21956/openreseurope.21698.r64700

**Published:** 2025-12-11

**Authors:** Tri Wintolo Apoko

**Affiliations:** 1 English Education, Universitas Muhammadiyah Prof Dr Hamka, South Jakarta, South Jakarta, Indonesia

This paper has a good topic and potentially describes sufficient and good results and impacts. Methodologically, this study benefits comprehensive results as this employs a mixed-methods design. However, there are some improvements to make this paper better and present more results and discussion which are based on the research questions you addressed: (1) the students' situations and experiences in learning a second language while away from home; (2) the personal development while studying abroad and their future careers. Below are some points to revise:

 1. Please include EU-funded programs and the local context in the introduction section

 2. In the literature review section, you may need to divide into two or three sub-sections, indicating the necessities of study abroad program, language acquisition and personal growth in some contexts, regions, etc.

 3. In research design, please clarify if you use an explanatory or exploratory design for the mixed-methods.

 4. For the context and participants, you may need to use a table for the participant demography.

 5. Describe the data collection, and followed with the data analysis section. For the questionnaire, is it developed by yourself or modified or adapted from a reference. You may also need to try-out the questionnaire for the validity and its reliability.

 6. Is the data analyzed with G-form or descriptive analysis or inreferential analysis? Please ensure about this for quantitative data. Regarding the qualitative data, what kind of data analysis technique did you use?

 7. In result section, please refer to the research question 1 and 2 as you formulated in the introduction section. Start with quantitative data, and then combine with qualitative data. Make sure you narrate both data.

 8. In the discussion, also you may need to refer to RQ1 and RQ2.

 9. To have sufficient data of this paper, please add more updated references with DOI.

If applicable, is the statistical analysis and its interpretation appropriate?

Partly

Is the study design appropriate and is the work technically sound?

Partly

Is the work clearly and accurately presented and does it engage with the current literature?

Partly

Are the conclusions drawn adequately supported by the results?

Partly

Are sufficient details of methods and analysis provided to allow replication by others?

Partly

Are all the source data and materials underlying the results available?

Partly

Reviewer Expertise:

ICT in ELT; English Teaching Methodology; Professional Teacher Development

I confirm that I have read this submission and believe that I have an appropriate level of expertise to confirm that it is of an acceptable scientific standard, however I have significant reservations, as outlined above.

# Reviewer report: Reviewer response for version 1

**DOI:** 10.21956/openreseurope.21698.r64699

**Published:** 2025-12-09

**Authors:** Teuta Agaj Avdiu

**Affiliations:** 1 Faculty of Education, University of Gjilan, GJILAN, Kosovo (Serbia and Montenegro)

The study is clearly written, well-grounded in relevant literature, and uses an appropriate mixed-methods design, combining quantitative questionnaires and qualitative interviews to explore students’ experiences in depth. The methodological details provided are sufficient for replication, and the data and materials underlying the results are reportedly available, enhancing transparency. The statistical analyses and interpretations are appropriate to the research questions, and the integration of quantitative and qualitative findings is coherent and technically sound. Overall, the conclusions are well supported by the evidence presented, demonstrating convincingly that immersion-based study-abroad experiences have a significant impact on students’ linguistic, cognitive, and personal development.

If applicable, is the statistical analysis and its interpretation appropriate?

Yes

Is the study design appropriate and is the work technically sound?

Yes

Is the work clearly and accurately presented and does it engage with the current literature?

Yes

Are the conclusions drawn adequately supported by the results?

Yes

Are sufficient details of methods and analysis provided to allow replication by others?

Yes

Are all the source data and materials underlying the results available?

Yes

Reviewer Expertise:

Applied Linguistics

I confirm that I have read this submission and believe that I have an appropriate level of expertise to confirm that it is of an acceptable scientific standard.
